# Severe, Acute Watery Diarrhea in an Adult

**DOI:** 10.1371/journal.pntd.0000898

**Published:** 2010-11-30

**Authors:** Fahima Chowdhury, Ashraful Islam Khan, Abu Syed Golam Faruque, Edward T. Ryan

**Affiliations:** 1 Clinial Sciences Division, International Centre for Diarrhoeal Disease, Research, Bangladesh (ICDDR,B), Dhaka, Bangladesh; 2 Division of Infectious Diseases, Massachusetts General Hospital, Boston, Massachusetts, United States of America; 3 Department of Medicine, Harvard Medical School, Boston, Massachusetts, United States of America; 4 Department of Immunology and Infectious Diseases, Harvard School of Public Health, Boston, Massachusetts, United States of America; Emory University, United States of America

## Case Description

**Figure 1:**
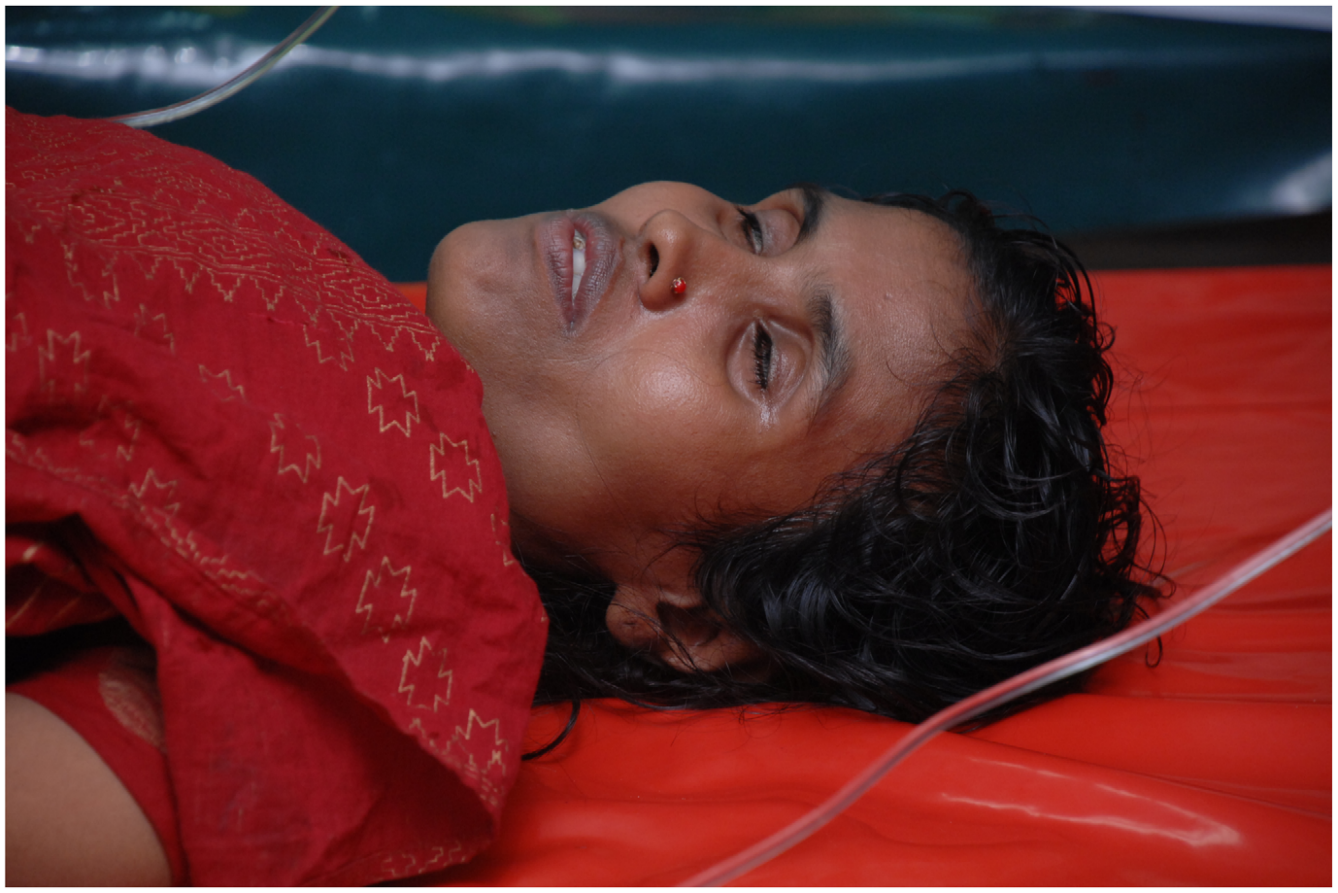
The patient on presentation.

**Figure 2:**
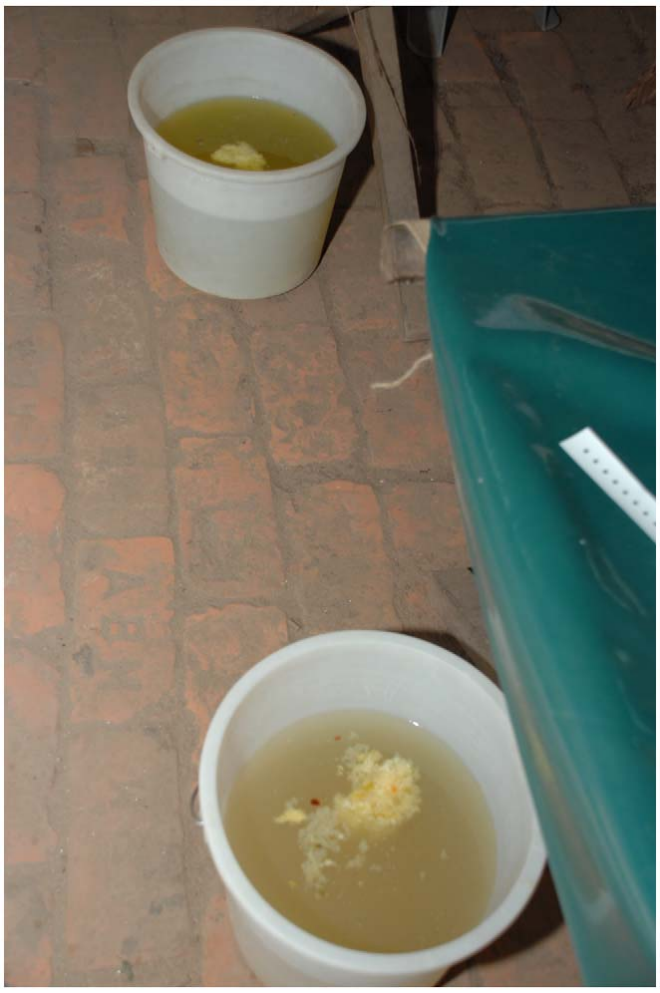
The patient's stool.

A 30-year-old woman presented to hospital in Dhaka, Bangladesh, with 10 hours of sudden onset of voluminous diarrhea and vomiting. Since onset, the patient had experienced seven episodes of diarrhea and three of vomiting, and ingested approximately 2 liters of oral rehydration solution at home. She lived in an informal settlement area of Dhaka with two young children and a husband who was a day-laborer. The family would often drink unboiled tap water stored in open large containers, and shared a toilet with approximately 20 other families. The patient's past medical history was unremarkable. On admission, no family member was suffering from severe diarrhea. On examination, the patient was lethargic and thirsty, and had sunken eyes, dry buccal mucosa, reduced skin turgor, deep and rapid breathing, and a feeble pulse (Figure 1). She had not urinated since onset of illness. Other systemic examination findings were normal. The patient's stool (Figure 2) had a fishy odor.

## Diagnosis

Cholera with severe dehydration leading to hypovolemic shock.

## Clinical Course

**Figure 3:**
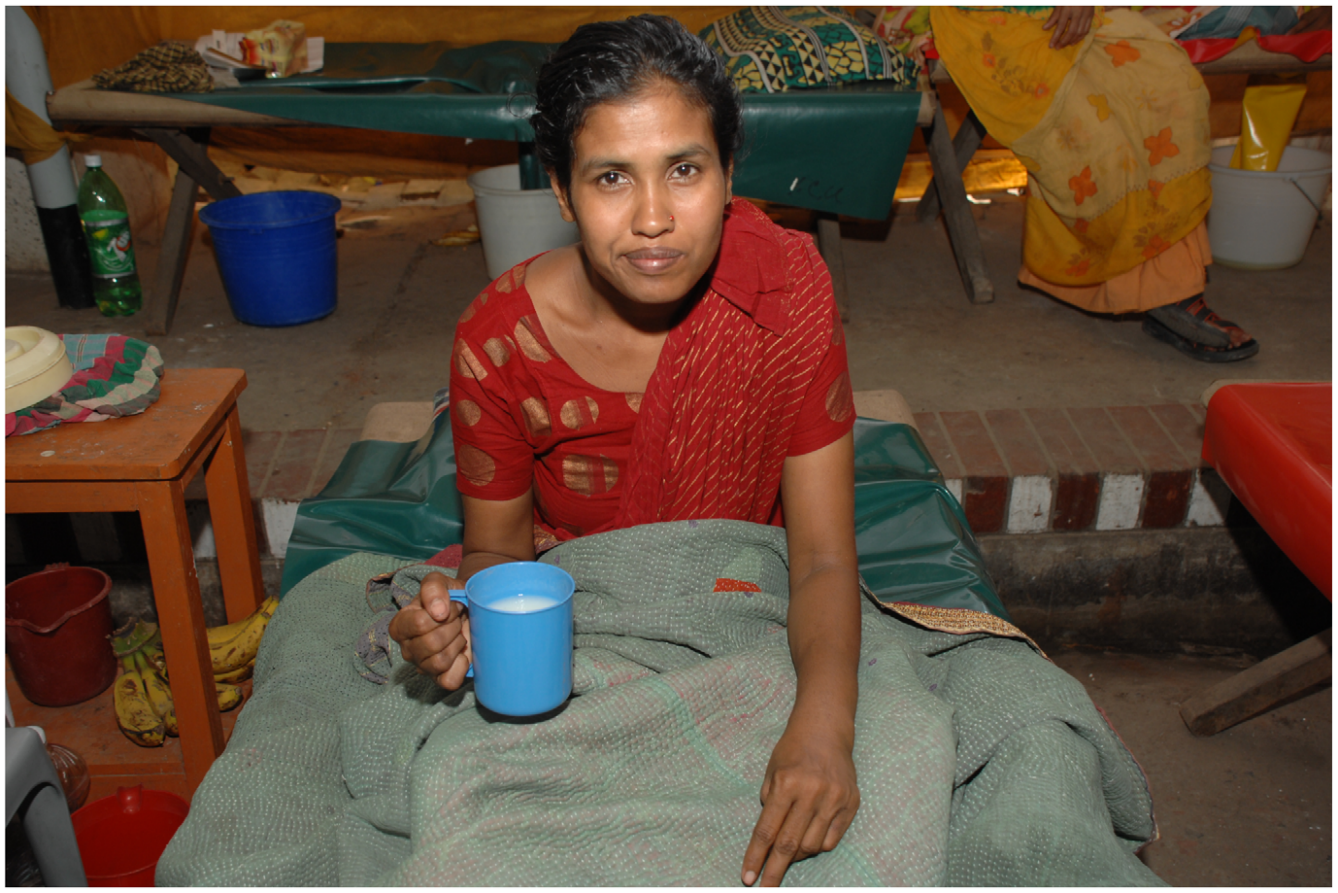
The patient 8 hours after starting rehydration therapy.

Upon arrival, the patient was categorized as severely dehydrated (>10% total body volume depleted), and immediately treated with intravenous infusion of cholera saline solution (Na^+133^, K^+13^, Cl^−98^, and acetate^−48^) at a rapid rate of 100 ml/kg over the first 3 hours (with an accelerated rate of 30 ml/kg over the first 30 minutes). The progress of rehydration therapy was assessed hourly. Due to lethargy, the patient was initially unable to consume appreciable amounts of oral liquid until approximately 3 hours after initiation of intravenous fluids, at which time the patient had received approximately 4 liters of intravenous fluid. As diarrhea continued, oral intake was continuously encouraged. Within 8 hours of arrival, the patient was alert and oriented with good skin turgor, moist mucus membranes, and a strong pulse. The patient began to void urine and was able to eat bread soaked in a cup of milk, and banana (Figure 3). The patient continued to drink 1 liter of oral rehydration solution (ORS) per hour to maintain fluid status while diarrhea continued. Darkfield microscopy disclosed “shooting star” movement. The patient was observed overnight, and then discharged and sent with home with ORS. Upon admission, the patient was treated with a single 1-gm oral dose of azithromycin.

## Discussion

This impoverished afebrile adult patient in Bangladesh had sudden onset of vomiting and voluminous rice-watery non-bloody diarrhea with a fishy smell, and presented with severe dehydration within a few hours of disease onset. Following rehydration, her clinical status quickly improved. All of these clinical features are compatible with cholera.

Diarrhea may be classified as watery (secretory) or dysenteric. Dysenteric diarrhea is usually associated with fever, macroscopic or microscopic presence of red and white blood cells in stool, and frequent passage of small volume mucoid stools. Dysentery is usually caused by enteropathogens that invade the intestinal epithelium such as *Shigella*, *Salmonella*, *Yersinia*, *Campylobacter*, and *Entamoeba histolytica*. Watery diarrhea is usually associated with non-invasive enteropathogens, is not usually associated with fever or blood in stool, and may be large volume. Severe watery diarrhea in a young child may be due to rotavirus (especially in the first one to two years of life), caliciviruses (including Norwalk virus and other noroviridae), enterotoxigenic *E. coli* (ETEC), and cholera. Sudden onset of a severe dehydrating diarrhea in individuals over the age of five should be highly suspicious for cholera, especially if more than one individual is affected in a resource-poor area. Diagnosis of *Vibrio cholerae* infection may be confirmed by seeing classic “shooting star” movement of organisms when looking at a cholera stool under a dark field microscope. A dipstick assay for rapid diagnosis use in resource-limited areas is available [1], and microbiological culture of stool or a rectal swab on selective media confirms the diagnosis, permitting typing and susceptibility testing.

Of the approximately 200 serogroups of *V. cholerae*, only serogroups O1 and O139 are associated with epidemic outbreaks of cholera. The world is currently experiencing its 7th cholera pandemic (caused by *V. cholerae* O1 El Tor). The vast majority of cholera cases are not reported to the World Health Organization (WHO), but it is estimated that at least 5–7 million individuals develop clinical cholera each year, resulting in over 100,000 deaths. *V. cholerae* is endemic in over 50 countries, and the global burden of disease is heaviest in South and Southeast Asia and sub-Saharan Africa. There have been recent epidemic outbreaks in Haiti, West Africa, and Zimbabwe. *V. cholerae* persists in the environment, and is associated with brackish estuarine water, especially in areas where fresh and salt water intermix. Humans become infected when they ingest water (or less likely, food) contaminated with *V. cholerae*, and the organisms becomes hyper-infectious following passage through the human intestine, facilitating explosive outbreaks and epidemics among immunologically susceptible populations [2].

Cholera is a disease of poverty, and is associated with war, displacement, an inability to obtain safe water, and an absence of proper sanitation facilities. Outbreaks have been associated with heavy rains and flooding [3]. In light of predicted regional increases in severe weather events, rising sea levels, and increasing flooding associated with global warming, as well as the ongoing urbanization of the world's population and growth of mega-cities lacking adequate infrastructure, the global burden of cholera may well increase over the next few decades.

Following ingestion, *V. cholerae* express cholera toxin (CT) within the intestinal lumen, resulting in increased cAMP levels in intestinal epithelial cells and pumping of Cl^−^ (and therefore Na^+^ and H_2_0) into the intestinal lumen and secretory diarrhea. Total flushing of the intestinal tract leads to “rice water” stool with a fishy odor. Although the classic clinical manifestation of cholera is a severe dehydrating diarrhea that can rapidly kill (cholera gravis), *V. cholerae* infection can cause a continuum of disease ranging from asymptomatic colonization to diarrhea of varying severity.

The treatment of individuals with cholera is predominantly based on fluid resuscitation and management. Individuals who die of cholera die of dehydration or complications of hypovolemic shock. Individuals with dehydrating diarrhea should be promptly assessed for level of dehydration (Box 1). Based on the level of dehydration and age or weight, individuals should be rapidly rehydrated to eu-volemia, and then adequate hydration maintained to replace ongoing fluid losses (Table 1). ORS, also called oral rehydration therapy or treatment (ORT), takes advantage of the fact that despite the action of CT, intestinal epithelial cells can still pump in electrolytes and water when sodium and glucose are presented at the same time (using a different pump than that affected by CT). Individuals who are unconscious or unable to ingest ORS by mouth can be treated either by passing ORS fluid down an inserted nasogastric tube, or by repleting fluids and electrolytes intravenously. ORS may be made using pre-packaged sachets containing salts and sugar and adding safe water, or at home by adding half a teaspoon of table salt and 4 tablespoons of table sugar to a liter of safe water, and encouraging potassium intake using bananas or green coconut water.

**Box 1. Assessing the Level of Dehydration**

**1. No dehydration, but diarrhea**

- Corresponds to <5% loss of total body weight

**2. Some or moderate dehydration**

Patient thirsty, dry mouth/tongue, no tears, sunken eyes, skin pinch slow to retract

- Corresponds to 5%–10% loss of total body weight

**3. Severe dehydration**

Patient unconscious, lethargic or floppy, weak pulse, unable to drink

Corresponds to >10% loss of total body weight

**Table 1:** Treatment (Based on Level of Dehydration).

**No Dehydration, but Diarrhea:**ORS replacement after each stool:• For children <2 years of age, give ¼–1/2 cup (50–100 ml) ORS to maximum of 0.5 liters per day.• For children 2–9 years of age, give 1/2–1 cup (100–200 ml) ORS to maximum of 1 liter per day.• For individuals 10 years of age and older, give as much ORS as wanted up to a maximim of 2 liters per day.• Reassess regularly, and follow stool and vomit output.

Some or Moderate Dehydration:						
ORS replacement to be given within first 4 hours						
Age	Less than 4 months	4–11 months	12–23 months	2–4 years	5–14 years	15 years
Weight	Less than 5 kg	5–<8 kg	8–<11 kg	11–<16 kg	16–<30 kg	30 kg or more
ORS	200–400 ml	400–600 ml	600–800 ml	800–1200 ml	1200–2200 ml	2200–4000 ml
•Reassess regularly, and follow stool and vomit output.						

**Severe Dehydration:**Administer intravenous fluid replacement with Ringer Lactate (or cholera saline) or, if not available, normal saline.• 100 ml/kg in first 3-hour period (slow to first 6-hour period when treating children less than 12 months of age)• Start rapidly (30 ml/kg within 30 min) and then slow down.Total amount per day: 200 ml/kg during the first 24 hours• Reassess regularly, and follow stool and vomit output.• Continue intravenous therapy until the patient is awake and able to ingest ORS and pulse is no longer weak.**Continue normal feeding during treatment**

Adapted from [8].

Following fluid management, the administration of antibiotics to individuals with cholera is only of secondary importance. The WHO recommends administration of antibiotics to cholera patients with severe dehydration only. Antibiotic administration is associated with a decreased volume of diarrhea, so in resource-limited settings antibiotic use will assist in conserving scarce resources, and antibiotic administration decreases the likelihood of secondary spread from a cholera patient to contacts. Unfortunately, *V. cholerae* O1 are becoming increasingly resistant to antimicrobial agents [4], [5]. Choice of an appropriate antibiotic should therefore consider regional susceptibility patterns. If regional strains are susceptible, young children and women of child-bearing age may be treated with erythromycin or azithromycin. Doxycycline and fluoroquinolones may be used to treat other individuals and/or based on susceptibility patterns.

A number of vaccines have been developed against cholera, and the most available vaccines globally are versions of an oral killed whole cell vaccine with or without addition of the non-toxic B subunit of CT [6], [7]. Oral administration of whole cell killed vaccines is associated with protective immunity that may be as high as 60%–90% immediately after vaccination, but requires more than one oral administration in immunologically naïve individuals, and protection wanes rapidly, decreasing to baseline within 6–36 months of vaccination.

**Key Learning Points**

- Diarrhea may be classified as watery or dysenteric.
- Severe watery diarrhea in an individual over 5 years of age should prompt consideration of cholera.
- Cholera is a disease of poverty and displacement.
- Individuals with cholera can die of dehydrating diarrhea within 12–24 hours of onset.
- The treatment of all individuals with diarrhea, but especially and emergently true for patients with cholera, includes rapid and simple assessment of the level of dehydration, followed by appropriate fluid replacement and management of ongoing fluid losses.
- Cholera patients with severe dehydration may also be treated with an appropriate antimicrobial agent.
- Although optimal prevention of cholera includes provision of safe water and adequate sanitation, such goals may not be feasible in the short or medium term for those most affected by cholera, suggesting that cholera vaccines may need to be used more widely.

## Supporting Information

Consent Form S1 (0.05 MB PDF)
