# Supplementary material for: Severe, Acute Watery Diarrhea in an Adult
Source: PLoS Negl Trop Dis. 2010 Nov 30;4(11):e898. doi: 10.1371/journal.pntd.0000898 (PMC2994913; doi:10.1371/journal.pntd.0000898)
Supplement: Consent Form S1 — (0.05 MB PDF) [file pntd.0000898.s001.pdf]

PLoS PUBLIC LIBRARY of SCIENCE

Consent Form for Publication in a PLoS Journal

I, the undersigned, give my consent for my photograph and/or case history to be published in a Public Library of Science (PLoS) Journal. I have seen and read the material to be published. I have discussed this consent form with ASG Faruque, who is an author of this paper, and I understand the following:

All PLoS journals are freely available on the web[1]. Hence, anyone anywhere in the world can read material published in them. Readers include not only doctors, but also journalists and other members of the public.

My name will not be published, and as far as possible all identifying features will be removed. However, it is not possible to ensure complete anonymity, and someone may be able to recognize me.

I understand that under the license which the PLoS uses (the Creative Commons Attribution License[2]) material published in PLoS journals can be redistributed freely and used for any legal purpose, including translation into other languages and commercial uses. I also understand that signing this consent form does not remove my rights to privacy.

Name ______________________________

Date ______________

Signed ______________________________

Author Dr. A.S.G. Faruque

Date 19 April 2009

Signed ASG Faruque

[1]PLoS Journals: http://www.plos.org/journals/

[2]Creative Commons Attribution License: http://creativecommons.org/licenses/by/2.5/

Please complete this form, obtain the patient's signature, and file in case notes.

The manuscript reporting this patient's details should state that consent to publication was obtained from the patient.

The Bangla translation is identical to English version of the consent form.

A M Khan
19.4.2009
DR. ALI MIRAJ KHAN
[illegible]
ICDDR,B

পিএলওএস

(পাবলিক লাইব্রেরী অফ সাইন্স)

পিএলওএস জার্নালে লেখা প্রকাশিত করবার জন্য সম্মতিপত্র

“পাবলিক লাইব্রেরী সাইন্স” নামক জার্নালে আমি লিখিত দস্তখতের মাধ্যমে আমার ছবি এবং রোগের বর্ণনা ছাপানোর জন্য সম্মতি দিয়েছি। যা ছাপানো হবে তা আমি দেখেছি এবং পড়েছি। আমি এই সম্মতিপত্র সম্মন্ধে এবং এই লেখার লেখক ডাক্তার এ এস জি ফারুক এর সাথে আলোচনা করেছি এবং নিম্নের বিষয়গুলি বুঝেছি ঃ

সকল "পাবলিক লাইব্রেরী সাইন্স” জার্নাল বিনামূল্যে ইন্টারনেটের ওয়েবসাইটে পাওয়া যায়। পৃথিবীর যে কোন জায়গা থেকে যে কেউ এই লেখা ওয়েব সাইটে গিয়ে পড়তে পারবে। যারা এই লেখা পড়বে তারা শুধু ডাক্তারই নন, সাংবাদিক অথবা সাধারন জনগনের যে কেউ হতে পারেন।

এই লেখায় আমার নাম প্রাকশিত হবেনা এবং যতটুকু সম্ভব আমাকে সনাক্ত করার সবকিছুই এখান থেকে সরিয়ে নেয়া হবে। যাই হোক যদিও আমাকে সনাক্ত না করার জন্য সকল পদক্ষেপ নেয়া হবে, কেউ হয়তবা আমাকে সনাক্ত করতেও পারে।

আমি আরও বুঝেছি যে পাবলিক লাইব্রেরী সাইন্স জার্নাল যে লাইসেন্স ব্যবহার করে (এইক্ষেত্রে মূল স্বত্বাধিকারীর লাইসেন্স) তার ফলে ছাপানো লেখা যা পিএলওএস জার্নালে আছে তা অনায়াসে বিতরন এবং নৈতিকভাবে ব্যবহার করা হবে। এমনকি অন্য ভাষায় রূপান্তরিত করে বা ব্যবসায়িক কাজেও ব্যবহার হতে পারে। আমি এও বুঝি যে, এই সম্মতিপত্রে দস্তখত আমার ব্যক্তিগত স্বার্থকে ব্যাহত করবে না।

নাম :---চান বানু---

তারিখ: ---১৯ - ৪ - ২০০৯---

দস্তখত :---চান বানু---

লেখক :---Dr. ASG Faruque---

তারিখ : ---19-4-2009---

দস্তখত: ---ASG Faruque---
